# A Twin Study on the Relation Between Positive Mental Health and Biological Aging

**DOI:** 10.3390/ijms27093729

**Published:** 2026-04-22

**Authors:** Corrado Fagnani, Angelo Picardi, Emanuela Medda, Miriam Salemi, Cristina D’Ippolito, Ester Siniscalchi, Francesca Salani, Giorgia M. Varalda, Francesca Marcon

**Affiliations:** 1 Center for Behavioral Sciences and Mental Health, Istituto Superiore di Sanità, 00161 Rome, Italy; angelo.picardi@iss.it (A.P.); emanuela.medda@iss.it (E.M.); miriam.salemi@iss.it (M.S.); cristina.dippolito@iss.it (C.D.); 2 Unit of Mechanisms/Biomarkers/Models, Department of Environment and Health, Istituto Superiore di Sanità, 00161 Rome, Italy; ester.siniscalchi@iss.it (E.S.); francesca.salani@iss.it (F.S.); giorgia.varalda@guest.iss.it (G.M.V.); francesca.marcon@iss.it (F.M.)

**Keywords:** aging, mental health, twins

## Abstract

Positive mental health (PMH) has recently become a key topic in biomedical research. Previous studies have explored the correlation between biological and psychological measures, but only a few have focused on the relationship between PMH and aging. This study aimed: (i) to explore the association between PMH and biological aging; (ii) to determine if and to what extent the observed association could be explained by shared genetic and environmental effects. A total of 401 twins (age 19–81 years, 32% male) from the Italian Twin Registry were recruited, and the twin study design was applied. A self-report psychological test battery was used to evaluate several PMH components. Blood samples were collected from participants to determine telomere length (TL) and mitochondrial DNA copy number (mtDNAcn). TL was negatively associated with attachment anxiety (r = −0.11, *p* = 0.037). A bivariate twin model provided heritability estimates of 0.14 (95% CI 0.001–0.43) for TL and 0.32 (0.16–0.45) for attachment anxiety, and a substantial negative genetic correlation [r_g_ = −0.55 (−1.00–0.00)] between them. Under the limitations of a cross-sectional study with a self-report wellbeing assessment, these results suggest that anxiety in the relationship with a partner may contribute to accelerated TL shortening, and shared genetic factors may underlie this link.

## 1. Introduction

Positive mental health (PMH) has recently become a key topic in scientific, particularly biomedical, research. In fact, it has been recognized that a deeper knowledge about the factors that affect PMH could represent a promising avenue for a better understanding and management of mind–body comorbidity. Defining PMH is a complex task that requires a multifaceted approach. An authoritative review has listed a number of models that are relevant to the construct, i.e., above-normal functioning, the presence of multiple human strengths, maturity, the dominance of positive emotions, high social and emotional intelligence, subjective wellbeing, and resilience [1]. Previous studies have explored the correlation between biological and psychological measures [2], but only a few were targeted on the relationship between PMH and aging [3,4]. Notably, a research indicated that posttraumatic stress disorder (PTSD) symptoms could be associated with accelerated aging, and positive mental health may have a role in moderating this link [5]. The few available studies on the association between psychological status and aging considered only specific aspects of PMH, whereas some of the relevant PMH components outlined above have been entirely overlooked. Moreover, evidence on the biological basis of the relationship between PMH and aging is still limited [3,4]. There are several plausible biological pathways that may account for a relationship between PMH and biological aging. Lower levels of PMH dimensions are associated with increased levels of psychological stress, and chronic stress may accelerate telomere attrition by triggering high oxidative stress that directly damages telomeric DNA, reducing telomerase activity, raising cortisol levels, and causing chronic inflammation. There is considerable evidence that oxidative stress can contribute to telomeric attrition [6]. A seminal study on healthy premenopausal women found that psychological stress is significantly associated with higher oxidative stress, lower telomerase activity, and shorter telomere length in peripheral blood mononuclear cells [7]. Indeed, glucocorticoids, reactive oxygen species, mitochondria, and inflammation play key roles in mediating the relationship between psychological stress and telomere maintenance [8]. Insecure attachment, in particular, is known to lead to difficulties in stress regulation, and the resulting chronic elevated stress response may increase inflammation and oxidative stress, which accelerates telomere attrition. Recent studies reported that high attachment anxiety was associated with shorter length of telomeres through high self-reported stress [9], and that attachment avoidance was associated with shorter telomere length [10]. Because fluctuations in telomere maintenance and mitochondrial function often mirror the systemic impact of the psychological state [11,12], these molecular changes can be used as key indicators of biological age. By tracking these molecular markers, researchers can identify shifts in aging rate and evaluate the risk of age-associated diseases regardless of chronological age [13,14].

In our study, we aimed: (i) to explore the association between several PMH components and specific biomarkers of biological age, which are more reliable than chronological age to investigate the effects associated with aging [13,15]; (ii) to determine if and to what extent the observed association could be explained by genetic and environmental correlations between the targeted psychological and biological measures.

For these purposes, we recruited a general population sample of adult twins enrolled in the Italian Twin Registry (ITR) [16] and applied the genetically informative twin study design, which has never been used previously in this line of research. Based on the comparison between monozygotic (MZ) twins, genetically identical, and dizygotic (DZ) twins, sharing 50% of their genetic background, the design allows for the estimation of genetic and environmental contributions to the expression of a given phenotype (or disease) and the co-expression (or co-morbidity) of multiple phenotypes (or diseases) [17].

## 2. Results

The main socio-demographic variables (i.e., sex, age, education, marital status, occupation) did not differ between subjects participating in this study and the reference ITR population resident in the targeted area. Routine check-up analyses showed that study subjects were in good health, with blood parameters (i.e., full blood count, glucose, creatinine, total proteins, GOT, GPT, cholesterol, triglycerides) generally within the normal ranges, apart from very few borderline exceptions.

PMH sample data were almost complete, with missingness below 5% for all scales. Reliability analyses revealed adequate internal consistency of these scales, with Cronbach’s alphas ranging from 0.78 to 0.93. Physical activity data were available for 334 out of 401 subjects (17% missingness); for these subjects, the level of activity as measured by metabolic equivalents (METs) was positively correlated with self-esteem score (r = 0.16, *p* = 0.003), resilience (r = 0.17, *p* = 0.002), empathy (r = 0.11, *p* = 0.05), positive affectivity (r = 0.18, *p* = 0.001), and Ryff’s wellbeing (r = 0.11, *p* = 0.036), while it was negatively correlated with an anxious attachment to a partner (r = −0.12, *p* = 0.041) and with difficulties in emotion recognition or regulation (r = −0.13, *p* = 0.021). No significant correlations were detected between METs and any of the two aging biomarkers measured; however, a positive association with TL was suggested (r = 0.07), which did not reach statistical significance (*p* = 0.18). Physical activity was not considered in subsequent analyses to avoid substantial sample size reductions due to the large missingness fraction for this variable.

**Figure 1:**
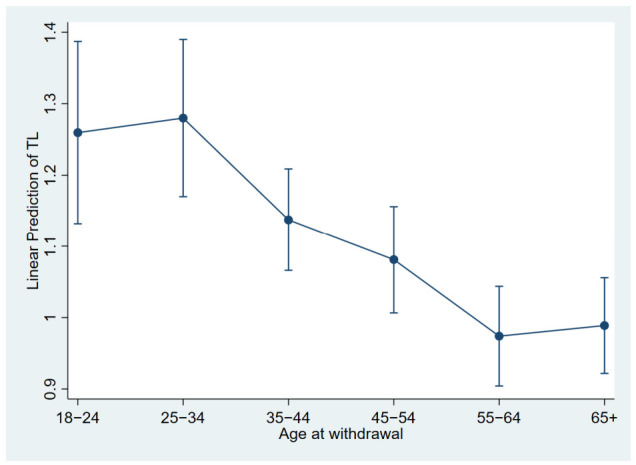
Association between TL and age at withdrawal. The figure reports the predictive margins under the linear regression of TL on age at withdrawal, sex and mtDNAcn. Vertical bars represent 95% confidence intervals. TL: telomere length.

As for aging biomarkers, TL and mtDNAcn were assessed for 391 and 390 out of 401 subjects respectively, resulting in less than 2.5% missingness for both these measures. TL was positively correlated with mtDNAcn (age- and sex-adjusted correlation: r = 0.14, *p* < 0.01), and both measures were higher in females compared to males (*p* = 0.012 for TL, *p* = 0.015 for mtDNAcn). With respect to relatedness to aging, TL decreased with age at withdrawal independently of sex and mtDNAcn (Figure 1), while a positive association with age was detected for mtDNAcn regardless of sex and TL (r = 0.12, *p* = 0.018), suggesting alterations in telomere maintenance and mitochondrial biogenesis related to the process of aging. Since the age-related trend for mtDNAcn was less clear than for TL, subsequent analyses were centered on TL as the main aging biomarker, while mtDNAcn was considered among the relevant adjusting variables.

In individual-level analysis, a linear regression model of TL on PMH dimensions showed that TL was negatively associated with an anxious attachment style as assessed by the ECR questionnaire, and this association was independent of sex, age, zygosity, mtDNAcn, and personality characteristics as evaluated by the BFI questionnaire (beta = −0.002, *p* = 0.049, corresponding to a partial correlation between TL and anxious attachment of: r = −0.11, *p* = 0.037). This result drew attention to the link between higher anxiety in the relationship with a partner and possible accelerated cellular aging, which deserved further investigation.

**Figure 2:**
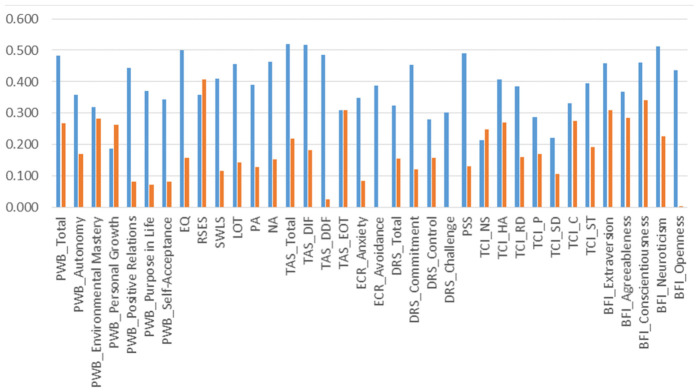
Intrapair correlations for PMH scale scores by zygosity. Blue and orange bars indicate correlations in monozygotic (MZ) and dizygotic (DZ) twin pairs, respectively. Correlation estimates are adjusted by sex and age at withdrawal. PWB_Total: total psychological wellbeing scale. PWB_Autonomy: “autonomy” PWB subscale. PWB_Environmental Mastery: “environmental mastery” PWB subscale. PWB_Personal Growth: “personal growth” PWB subscale. PWB_Positive Relations: “positive relations” PWB subscale. PWB_Purpose in Life: “purpose in life” PWB subscale. PWB_Self-Acceptance: “self-acceptance” PWB subscale. EQ: empathy quotient. RSES: Rosenberg self-esteem scale. SWLS: satisfaction with life scale. LOT: life orientation test. PA: positive affect scale. NA: negative affect scale. TAS_Total: total Toronto alexithymia scale. TAS_DIF: “difficulties in identifying feelings” TAS subscale. TAS_DDF: “difficulties in describing feelings” TAS subscale. TAS_EOT: “externally oriented thinking” TAS subscale. ECR_Anxiety: “anxious attachment” ECR (experiences in close relationships) subscale. ECR_Avoidance: “avoidant attachment” ECR subscale. DRS_Total: total dispositional resilience scale. DRS_Commitment: “commitment” DRS subscale. DRS_Control: “control” DRS subscale. DRS_Challenge: “challenge” DRS subscale. PSS: perceived stress scale. TCI_NS: “novelty seeking” TCI (temperament and character inventory) subscale. TCI_HA: “harm avoidance” TCI subscale. TCI_RD: “reward dependence” TCI subscale. TCI_P: “persistence” TCI subscale. TCI_SD: “self-directedness” TCI subscale. TCI_C: “cooperativeness” TCI subscale. TCI_ST: “self-transcendence” TCI subscale. BFI_Extraversion: “extraversion” BFI (big five inventory) subscale. BFI_Agreeableness: “agreeableness” BFI subscale. BFI_Conscientiousness: “conscientiousness” BFI subscale. BFI_Neuroticism: “neuroticism” BFI subscale. BFI_Openness: “openness” BFI subscale.

Intrapair correlation analysis applied to PMH dimensions and stratified by zygosity showed substantially higher sex- and age-adjusted correlations in MZ compared to DZ twins for the scores of almost all PMH scales, with a few exceptions (Figure 2 and Supplementary Table S1). The same analysis, applied to TL and adjusted by sex, age, mtDNAcn, and personality BFI factors, provided a slightly higher correlation for MZ twins (r_MZ_ = 0.72) compared to DZ twins (r_DZ_ = 0.65). These findings suggested sizeable genetic influences for most psychological traits, and a weak genetic load with major shared-familial and unique (individual-specific) environmental effects for TL dynamics.

In the MZ intrapair-difference regression model, adjusted by (intrapair difference in) mtDNAcn and BFI factors (sex and age were no longer considered as covariates because they were fully matched between MZ twins), the association between (intrapair difference in) TL and (intrapair difference in) anxious attachment completely disappeared (beta = −0.0002, *p* = 1.00). This suggested a role of genetic or shared-familial confounding in the individual-level association between TL and anxious attachment, and therefore indicated possible genetic or shared-familial correlation between TL and anxious attachment, which we estimated in subsequent correlated-factor twin analyses.

**Figure 3:**
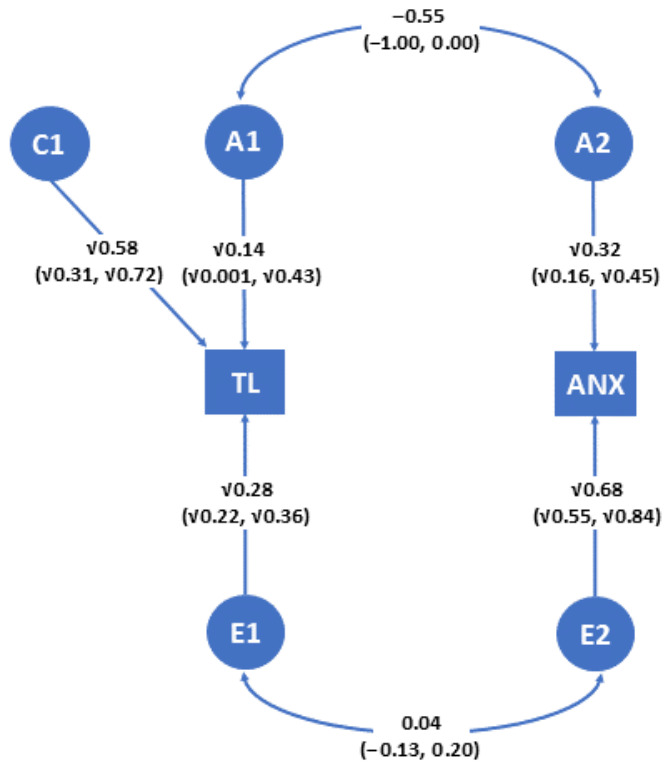
Bivariate correlated-factor model for the association between telomere length and anxious attachment. The model was fitted to sex-, age-, mtDNAcn-, and BFI (personality)-adjusted correlations. TL: telomere length. ANX: anxious attachment. A1,A2: additive genetic effects on the two phenotypes. C1: shared-familial effects on TL. E1,E2: unique environmental effects on the two phenotypes. For the sake of simplicity, the model is depicted for one twin only. By squaring coefficients on single-headed arrows, the genetic and environmental proportions of variance of each phenotype can be obtained. Coefficients on double-headed arrows indicate genetic and environmental correlations between the two phenotypes. Numbers in parentheses represent 95% confidence intervals of estimates.

A bivariate correlated-factor model for TL and anxious attachment, fitted to sex-, age-, mtDNAcn-, and BFI-adjusted correlations in MZ and DZ groups, and including (i) additive genetic effects for both TL and anxious attachment, (ii) shared-familial effects only for TL, and (iii) unique environmental effects for both TL and anxious attachment, provided heritability estimates of 0.14 for TL and 0.32 for attachment anxiety, and a substantial negative genetic correlation (r_g_ = −0.55) between them (Figure 3).

## 3. Discussion

This is the first study that uses the genetically informative twin design to unravel the link between PMH and biological aging. Our results suggest that, in the Italian general adult population, an anxious attachment style in the relationship with a partner may be associated with accelerated cellular aging as assessed by TL, and this association may be explained, at least in part, by genetic influences in common for attachment anxiety and biological aging. These findings may be of relevance in genetic research; for example, by directing the search of aging-related genetic factors towards those genetic factors that have already been identified for anxiety-related phenotypes [18]. Furthermore, the results may open novel opportunities for intervention programs. For example, the promotion of non-anxious, secure attachment styles, besides improving the quality of romantic relationships and the wellbeing of involved partners, may also reduce the effects of accelerated biological aging. Also, it may be worthwhile to monitor individuals for possible accelerated biological aging in the presence of anxiety symptoms—particularly in attachment styles—not only in the same individuals but also in their family members.

Our exploratory results must be interpreted with caution and need to be confirmed by replication studies that should consider alternative PMH models and aging biomarkers. In particular, it could be valuable to integrate biological analyses using epigenetic markers of aging, such as methylation levels of selected genes mirroring the biological clock, together with proteomic profiling [19,20]. At the same time, a certain degree of confidence may be placed in these results, as our relatively large, registry-based twin sample may have provided at least partial reassurance against major selection biases. Indeed, the fact that the main socio-demographic variables did not differ between subjects participating in this study and the reference ITR population resident in the targeted area, along with the expected observations of the higher correlation in MZ compared to DZ twin pairs for the most PMH traits, as well as the positive correlation between TL and mtDNAcn and the female advantage for both these aging biomarkers, all speak in favor of the adequate external validity of our study. It is also noteworthy that the negative correlation we found between TL and anxious attachment is in line with the results of a relatively recent meta-analysis on the relationship between TL and anxiety [21]. As the only exception, we failed to detect a clear association between physical activity and aging biomarkers. The weak positive association we found between METs and TL did not reach statistical significance, probably due to power issues. Furthermore, our study was not specifically designed to estimate the aging-related effects of physical activity; therefore, the low-medium levels of physical activity measured in our sample were probably not sufficient to modulate the aging biomarkers investigated herein. Among the physiological changes possibly linking chronic psychological stress to altered telomere biology, the level of glucocorticoids has been proposed as a key stress mediator. On one side, stress is associated with an increased plasma level of glucocorticoids due to the activation of the hypothalamic–pituitary–adrenal axis [22,23]. On the other side, glucocorticoids may trigger an increase in the production of reactive oxygen species (ROS), whose role in telomere shortening is well established [24], affecting metabolic rates and mitochondrial activity [25] as well as directly acting on the redox balance [26,27]. Glucocorticoids also have a pivotal role in inflammatory response, with high levels of glucocorticoids associated with alterations in the expression of pro- and anti-inflammatory genes [28]. Notably, the critical role of chronic inflammation on telomere length has been consistently reported [29,30]. Therefore, multiple and interconnected pathways seem to be involved in the transduction of psychological stress into physiological and cellular changes affecting telomere maintenance [8]). It is of note, however, that the conceptualization of telomere length—whether it serves as a biomarker for chronological senescence or as a proxy for the psychological distress of pathology—remains a subject for further debate within the broader framework of lifespan development. The present investigation limits our ability to resolve this debate, as longitudinal data would be required to answer this pivotal question, rather than the cross-sectional design applied herein [31].

In addition, the independent effect of aging on telomere length and mitochondrial function highlighted in the present study suggests that age-related cellular processes can also modulate telomeres and mitochondria biology through distinct, autonomous pathways, despite the strong correlation between these two biomarkers [32].

A further important message of our study is that molecular research in general, and biological aging research in particular, may benefit from incorporating psychological variables in the analysis of biological processes, as these variables may play contributing roles due to the well-documented mind–body connection. This appears even more true when one considers that molecular research is often concerned about effects of modest size; such effects may easily be masked by confounding mechanisms in which psychological components may also be involved. With respect to this study, we would have failed to detect the weak inverse association of TL with anxious attachment and the subtle genetic influences on TL if we had not considered personality factors among the relevant covariates. From this point of view, it is desirable that future molecular studies on aging will exploit psychological and mental health data in their search for significant yet small signals.

Regarding the weak heritability found for TL, although not totally new [33], it is generally in contrast with prior literature showing moderate to high estimates for genetic effects on this biomarker. One possible explanation is that our study was conducted during the COVID-19 pandemic, whose global population impact in terms of stress-related experiences may have inflated the estimate of the shared environmental component of telomere dynamics, thus partially masking the contribution of heritable factors.

As a main limitation of our study, the self-reported nature of collected data may raise concerns about their reliability. However, internal consistency analyses showed an adequate reliability of the PMH data. In this respect, it may also be worth highlighting that the filled-in PMH questionnaires were routinely double-checked when they were brought back by the participants at the reference laboratory, and incompleteness or major incongruities were promptly resolved on that occasion. A second study weakness may be the cross-sectional design, which precluded causal inference, and also prevented us from investigating the individual longitudinal changes in PMH and the impact of these changes on TL dynamics. Regarding causal inference, this limitation may have been mitigated by the use of the MZ intrapair-difference model, which represents an optimal surrogate for experimental designs, and can provide strong indications about causality even in the context of observational, cross-sectional studies. As a final remark, we should acknowledge that our analytical strategy treated the several PMH components as separate entities, which may have led to us overlooking the effects of the underlying correlational structure of PMH traits. For this reason, future analyses could benefit from modeling PMH as a higher-order latent construct, which may better capture the composite contribution of the various PMH dimensions, thus potentially providing enhanced opportunities to detect associations with aging biomarkers.

## 4. Materials and Methods

### 4.1. Participants

The reference population was represented by adult twins residing in Rome and its province, who were previously enrolled in the ITR. Study subjects were recruited following a multi-step/multi-method approach, using email, postal mail, or SMS as contact tools to send the study invitation along with general information on study objectives and procedures to consecutive subgroups of eligible subjects (N 3222). Study documents including an information letter, an informed consent form, and an extensive self-report PMH test battery were sent by post only to those subjects potentially volunteering to take part in the research. In case of willingness to participate, date and time of the blood sample withdrawal at the reference laboratory were fixed by telephone, and subjects were required to bring the filled-in questionnaires back to the ITR team on that occasion. The only exclusion criterion was a previous diagnosis of major medical or psychiatric diseases.

The target sample size (around 200 twin pairs) was defined a priori based on a trade-off between statistical power needs and space/personnel constraints of the external reference laboratory. Once the target sample size was reached, the invitation phase was stopped, and subsequent study phases were implemented in the final sample. A total of 401 twins from 199 intact pairs plus three unmatched twins (32% male; 58% MZ; mean age: 51 years; age range: 19–81 years), resident in Rome and its province, were recruited.

### 4.2. Assessment of PMH

The following self-report psychological test battery was administered to participants to assess PMH: (i) Ryff scales of Psychological Wellbeing (PWB) [34] and Empathy Quotient (EQ) [35]; (ii) Rosenberg Self-Esteem scale (RSES) [36], Satisfaction with Life scale (SWLS) [37], and Life Orientation Test (LOT) [38]; (iii) Positive and Negative Affect Schedule (PANAS) [39]; (iv) Toronto Alexithymia Scale (TAS-20) [40]; (v) Experiences in Close Relationships (ECR) questionnaire [41]; (vi) Dispositional Resilience Scale (DRS) [42]; (vii) Cohen’s Perceived Stress Scale (PSS) [43]. Furthermore, the Temperament and Character Inventory (TCI-125) [44] and the Big Five Inventory (BFI) [45] were administered, and the International Physical Activity Questionnaire (IPAQ) [46] was also used.

### 4.3. Samplings

Blood samples were collected from all participants by venipuncture at a reference laboratory external to the Istituto Superiore di Sanità (ISS), where the ITR was held. An aliquot of blood was used for clinical biochemistry analyses (i.e., full blood count, glucose, creatinine, total proteins, GOT, GPT, cholesterol, triglycerides) performed by the external laboratory. The results of these routine check-up analyses were provided for free to participants and allowed for a general evaluation of their health status. Remaining blood samples were transported to ISS within an hour to be processed for serum and plasma collection and peripheral blood mononuclear cell (PBMC) isolation, as described elsewhere [47]. Biological samples were appropriately stored in the ITR biobank.

#### 4.3.1. Telomere Length (TL)

TL was measured by quantitative real-time PCR (qRT-PCR) using DNA samples purified from whole blood. This method is based on the rationale that the amount of telomere signal per genome measured by qPCR represents the average TL in a given DNA sample. TL was quantified as the relative ratio of telomere (T) repeat copy number to a single copy gene (S), called the T/S ratio, in experimental samples using standard curves. The 36B4, encoding acidic ribosomal phosphoprotein P0, was used as the control single copy gene needed to quantify input genomic DNA and to normalize the signal from the telomere reaction. The primer sequences for telomere amplification were:

(forward TelF) 5′-GGTTTTTGAGGGTGAGGGTGAGGGTGAGGGTGAGGGT-3′;

(reverse TelR) 5′-TCCCGACTATCCCTATCCCTATCCCTATCCCTATCCCTA-3′.

The primer sequences for the amplification of the reference gene 36B4 were:

(forward 36B4F) 5′CAGCAAGTGGGAAGGTGTAATCC3′;

(reverse 36B4R) 5′CCCATTCTATCATCAACGGGTACAA3′.

The reaction was carried out in triplicate. The PCR master mix included 10 µL of SensiFAST SYBR Hi-ROX master mix (Bioline Meridian Bioscience, London, UK), 2 µL of forward primer and 2 µL of reverse primer (working solution 1 µM; final concentration 100 nM), 5 µL of genomic DNA (working solution 4 ng/µL; final concentration 20 ng/well), and purified water to a total volume of 20 µL. A standard curve and a negative control (no DNA template) were included in each experiment. For the standard curve, a reference DNA sample was diluted serially to produce six final concentrations (20, 10, 5, 2.5, 1.25, 0.625 ng/well). The PCR cycling conditions for both amplicons were 95 °C for 20 s, followed by 40 cycles at 95 °C for 3 s and 60 °C for 30 s. The specificity of the PCR reaction was checked via the analysis of melting curves obtained at the end of each PCR. RT-PCRs were performed using the ABI Prism 7500 Sequence Detection System (Applied Biosystems, Monza, MB, Italy). Fluorescence was analyzed with the ABI Prism 7500 FAST SDS software, version 2.0 (2010), to quantify PCR products for each sample based on the standard curve. The resulting T/S ratio represented the average TL per genome. The same position for each sample (i.e., standard curve, test samples) was maintained on the two plates (telomere and 36B4 reactions).

#### 4.3.2. Mitochondrial DNA Copy Number (mtDNAcn)

Samples of DNA were diluted to obtain the working concentration of 4 ng/µL to be used for each experimental point carried out in triplicate. The amount of mitochondrial DNA (mtDNA) was measured using quantitative polymerase chain reaction (qRT-PCR) to determine the levels of the nuclear gene HBG (human beta-globin) and the mitochondrial gene ND1. The amplification of HBG was obtained using the following primers:

(forward) 5′-GAAGAGCCAAGGACAGGTAC-3′ and (reverse) 5′-CAATTCATCCACGTTCACC-3′,

while for ND1, the primers were as follows:

(forward) 5′-AACATACCCATGGCCAACCT-3′ and (reverse) 5′-AGCG-AAGGGTTGTAGTAGCCC-3′.

The amplification reaction contained 0.6 µL of each primer (working solution 10 µM; final concentration 300 nM), 10 µL of SYBR Green PCR Master Mix (Bioline Meridian Bioscience, London, UK), 5 µL of genomic DNA (working solution 4 ng/µL; final concentration 20 ng/well), and purified water to a total volume of 20 µL. A standard curve, a negative control (no DNA template), and a reference sample of genomic DNA were included in each experiment. For the standard curve, a sample of genomic DNA was diluted serially to produce five final concentrations (20, 10, 5, 2.5, 1.25 ng/well). The PCR cycling conditions for both amplicons were 95 °C for 10 min, followed by 40 cycles at 95 °C for 15 s and 60 °C for 1 min. The specificity of the PCR reaction was checked via the analysis of melting curves obtained at the end of each PCR. RT-PCR was performed using the ABI Prism 7500 Sequence Detection System (Applied Biosystems, Monza, MB, Italy). Fluorescence was analyzed using the ABI Prism 7500 FAST SDS software, version 2.0 (2010), to quantify PCR products for each sample based on the standard curve. The amount of mtDNA was obtained by the ratio between the copy number of the mitochondrial gene ND1 and the nuclear gene HBG. The ratio of mtND1/HBG was normalized using the reference sample of genomic DNA. The same position for each sample (i.e., standard curve, reference sample, test samples) was maintained on the two plates (nuclear HBG and mitochondrial ND1 reactions).

### 4.4. Statistical Analyses

As a preliminary step, the internal consistency reliability of the PMH scales was inspected using Cronbach’s alpha statistic (≥0.70 minimum acceptable value). In subsequent steps, different modeling strategies were applied.

First, twins were considered as individual subjects, and linear regression models were fitted to estimate the association of aging biomarkers (i.e., TL and mtDNAcn) and PMH traits, adjusting standard errors of the estimates for the non-independence of data within twin pairs.

Second, within-pair correlations for aging biomarkers and PMH traits were estimated and compared between MZ and DZ pairs to gain general insights about the role of genetic and environmental factors in the expression of these characteristics [17].

Third, for those associations emerging from the individual-level regression analysis, the monozygotic (MZ) intrapair-difference model was applied. According to this model, if the association persisted when regressing the within-pair difference in the aging biomarker on the within-pair difference in the PMH trait (i.e., when optimally controlling for genetic and shared-familial confounding factors), then the association was interpreted as being compatible with a causal link between the PMH trait and the aging biomarker. On the contrary, if the association vanished, then a causal link was excluded in favor of confounding by genetic or environmental factors simultaneously impacting on the aging biomarker and the PMH trait (i.e., genetic or environmental correlation between the aging biomarker and the PMH trait) [48].

Fourth, when genetic or environmental confounding was suggested by the MZ intrapair-difference model, bivariate correlated-factor twin models were fitted to correlations in MZ and DZ groups to estimate the genetic and environmental correlations between the aging biomarker and the PMH trait, as well as their heritability. Heritability answers the question regarding to what extent individual differences in a trait are explained by genetic factors; the genetic (environmental) correlation between two traits answers the question concerning to what extent genetic (environmental) factors acting on one of the traits overlaps with those acting on the other trait. In these models, latent factors for additive genetic, shared (i.e., familial) environmental, and unique (i.e., individual-specific) environmental influences on the aging biomarker and the PMH trait were included as sources of their variance and covariance, and were allowed to correlate between twins within pairs according to the degree of their genetic relatedness [49].

Stata software version 19 (StataCorp LLC, College Station, TX, USA) was used for all analyses except for correlated-factor twin modeling, which was performed by the Mx program (version 1.7.03) [50].

## 5. Conclusions

This study represents the first effort to unravel the connection between mental health and cellular aging by the twin design. Under the limitations of a cross-sectional study based on self-reported wellbeing information, our results suggest that anxiety in the relationship with a partner may be a relevant contributor in accelerated TL shortening; furthermore, shared genetic effects may be at the basis of this link.

If confirmed in future studies, these findings may contribute in directing the search for aging-related genetic factors towards those genetic factors already identified for anxiety-related phenotypes. The results may also provide a rationale for intervention programs aimed at buffering the effects of cellular aging by the promotion of secure attachment styles in romantic relationships, or by the anxiety-driven monitoring of individuals and their family members.

## Data Availability

The data presented in this study are available on request from the corresponding author. The original data are electronically stored at the Istituto Superiore di Sanità.

## Supplementary Materials

The following supporting information can be downloaded at: https://www.mdpi.com/article/10.3390/ijms27093729/s1.
