# Supplementary material for: A Twin Study on the Relation Between Positive Mental Health and Biological Aging
Source: Int J Mol Sci. 2026 Apr 22;27(9):3729. doi: 10.3390/ijms27093729 (PMC13164265; doi:10.3390/ijms27093729)
Supplement: Supplementary file 1 [file ijms-27-03729-s001.zip › ijms-4223836-supplementary.pdf]

Supplementary Table S1. Intrapair correlations for PMH (positive mental health) scales' scores by zygosity.

	MZ	p_{MZ}	DZ	p_{DZ}	p_{DIFF}
PWB_Total	0.483	0.0000	0.268	0.0070	0.0422
PWB_Autonomy	0.359	0.0000	0.170	0.0623	0.0813
PWB_Environmental Mastery	0.318	0.0002	0.283	0.0046	0.3961
PWB_Personal Growth	0.186	0.0227	0.264	0.0078	0.2868
PWB_Positive Relations	0.443	0.0000	0.081	0.2339	0.0034
PWB_Purpose in Life	0.371	0.0000	0.073	0.2565	0.0152
PWB_Self-Acceptance	0.344	0.0001	0.081	0.2339	0.0288
EQ	0.500	0.0000	0.158	0.0771	0.0038
RSES	0.358	0.0000	0.406	0.0001	0.3502
SWLS	0.409	0.0000	0.117	0.1466	0.0151
LOT	0.456	0.0000	0.144	0.0973	0.0087
PA	0.390	0.0000	0.128	0.1248	0.0263
NA	0.463	0.0000	0.154	0.0825	0.0090
TAS_Total	0.519	0.0000	0.218	0.0238	0.0078
TAS_DIF	0.516	0.0000	0.182	0.0499	0.0041
TAS_DDF	0.486	0.0000	0.026	0.4080	0.0003
TAS_EOT	0.310	0.0003	0.309	0.0021	0.4970
ECR_Anxiety	0.349	0.0001	0.084	0.2257	0.0276
ECR_Avoidance	0.387	0.0000	0.000	0.5000	0.0026
DRS_Total	0.324	0.0002	0.156	0.0797	0.1105
DRS_Commitment	0.453	0.0000	0.121	0.1384	0.0060
DRS_Control	0.280	0.0011	0.158	0.0771	0.1899
DRS_Challenge	0.303	0.0004	0.002	0.4929	0.0167
PSS	0.490	0.0000	0.132	0.1175	0.0029
TCI_NS	0.213	0.0107	0.248	0.0117	0.4001
TCI_HA	0.406	0.0000	0.270	0.0066	0.1460
TCI_RD	0.385	0.0000	0.160	0.0744	0.0471
TCI_P	0.288	0.0008	0.170	0.0623	0.1967
TCI_SD	0.221	0.0085	0.106	0.1706	0.2091
TCI_C	0.332	0.0001	0.275	0.0058	0.3336
TCI_ST	0.394	0.0000	0.193	0.0402	0.0651
BFI_Extraversion	0.458	0.0000	0.310	0.0021	0.1165
BFI_Agreeableness	0.367	0.0000	0.286	0.0043	0.2673
BFI_Conscientiousness	0.462	0.0000	0.341	0.0007	0.1611
BFI_Neuroticism	0.513	0.0000	0.227	0.0194	0.0108
BFI_Openness	0.437	0.0000	0.003	0.4893	0.0007

Correlations' estimates are adjusted by sex and age at withdrawal.

PWB_Total-total psychological wellbeing scale. PWB_Autonomy-"autonomy" PWB subscale. PWB_Environmental Mastery-"environmental mastery" PWB subscale. PWB_Personal Growth-"personal growth" PWB subscale. PWB_Positive Relations-"positive relations" PWB subscale. PWB_Purpose in Life-"purpose in life" PWB subscale. PWB_Self-Acceptance-"self-acceptance" PWB subscale. EQ-empathy quotient. RSES-Rosenberg self-esteem scale. SWLS-satisfaction with life scale. LOT-life orientation test. PA-positive affect scale. NA-negative affect scale. TAS_Total-total Toronto alexithymia scale. TAS_DIF-"difficulties in identifying feelings" TAS subscale. TAS_DDF-"difficulties in describing feelings" TAS subscale. TAS_EOT-"externally oriented thinking" TAS subscale. ECR_Anxiety-"anxious attachment" ECR (experiences in close relationships) subscale. ECR_Avoidance-"avoidant attachment" ECR subscale.

DRS_Total-total dispositional resilience scale. DRS_Commitment-“commitment” DRS subscale. DRS_Control-“control” DRS subscale. DRS_Challenge-“challenge” DRS subscale. PSS-perceived stress scale. TCI_NS-“novelty seeking” TCI (temperament and character inventory) subscale. TCI_HA-“harm avoidance” TCI subscale. TCI_RD-“reward dependence” TCI subscale. TCI_P-“persistence” TCI subscale. TCI_SD-“self-directedness” TCI subscale. TCI_C-“cooperativeness” TCI subscale. TCI_ST-“self-transcendence” TCI subscale. BFI_Extraversion-“extraversion” BFI (big five inventory) subscale. BFI_Agreeableness-“agreeableness” BFI subscale. BFI_Conscientiousness-“conscientiousness” BFI subscale. BFI_Neuroticism-“neuroticism” BFI subscale. BFI_Openness-“openness” BFI subscale.
MZ-monozygotic. DZ-dizygotic. p_{MZ}-one-tailed significance of MZ correlation. p_{DZ}-one-tailed significance of DZ correlation. p_{DIFF}-one-tailed significance of difference between MZ and DZ correlations.
